# The Impact of Employment and Economic Perception on Nutrition and Depression Among Cancer Survivors

**DOI:** 10.3390/healthcare13162075

**Published:** 2025-08-21

**Authors:** Guillermo Laporte-Estela, Manuel Rivera-Vélez, Paulette Ayala-Rodriguez, Gabriela Nichole Marrero-Quiñones, Zindie Rodriguez-Castro, Cynthia Cortes-Castro, Guillermo N. Armaiz-Pena, Eida M. Castro-Figueroa

**Affiliations:** 1 Ponce Research Institute, Ponce Health Scienes University, Ponce, PR 00716, USA; glaporte22@stu.psm.edu (G.L.-E.); manuelrivera@psm.edu (M.R.-V.); payala23@stu.psm.edu (P.A.-R.); gmarrero22@stu.psm.edu (G.N.M.-Q.); zrodriguez@psm.edu (Z.R.-C.); ccortes@psm.edu (C.C.-C.); garmaiz@psm.edu (G.N.A.-P.); 2 School of Behavioral and Brain Sciences, Ponce Health Sciences University, Ponce, PR 00716, USA; 3 Public Health Program, Ponce Health Sciences University, Ponce, PR 00716, USA; 4 School of Medicine, Ponce Health Sciences University, Ponce, PR 00716, USA; 5 School of Dental Medicine, Ponce Health Sciences University, Ponce, PR 00716, USA

**Keywords:** cancer patients, employment, economic perception, nutrition, depression, Puerto Rico, psychosocial oncology

## Abstract

Background: Cancer remains a leading cause of morbidity and mortality worldwide. In Puerto Rico, patients face additional burdens due to the structural inequalities affecting access to employment, nutritious food, and mental health services. This study examined the associations between employment status, perceived economic hardship, dietary behaviors, and depressive symptoms among 334 adult cancer patients in Puerto Rico. Methods: Using a cross-sectional design, participants provided sociodemographic data, dietary patterns, and self-reports of depression. Results: Statistical analyses revealed that full-time employment was associated with a higher consumption of low-nutritional-value foods (ρ = 0.157, *p* = 0.015) and significant differences in their consumption having a higher mean against unemployment were observed (mean ranks = 146.09 and 177.08, *p* = 0.010). A higher employment status also served as a protective factor against depression (*p* = 0.005). A higher body mass index (BMI) was linked to an increased risk of depression (*p* = 0.002), and perceived economic hardship was significantly associated with depression (OR= 0.54, *p* = 0.033). Conclusions: The findings underscore the necessity for comprehensive interventions that account for the synergistic effects of economic perception, employment, nutrition, and psychological well-being in cancer treatment in Puerto Rico.

## 1. Introduction

Cancer is one of the leading causes of morbidity and mortality worldwide. In the year 2022, approximately 20 million new cancer cases and 9.7 million related deaths were estimated globally, with an increasing trend attributed to population aging and modifiable risk factors such as diet, smoking, and physical inactivity [1,2]. These risk factors can be influenced by socioeconomic status, contributing to disparities in cancer incidence, diagnosis, and outcomes [3]. Additionally, cancer incidence is rising among younger adults in the United States, particularly for obesity-related cancers [4]. The diagnosis of cancer, beyond its physical effects, entails significant social, economic, and emotional challenges for affected individuals [5,6]. In the case of Puerto Rico, the repercussions of a cancer diagnosis extend beyond the physical and emotional toll. These challenges are further intensified by the longstanding socioeconomic disruptions that residents are currently facing. As a result, many individuals on the island experience substantial limitations in accessing essential resources such as stable employment, affordable and nutritious food, and timely mental health services. Such challenges create additional layers of vulnerability for cancer patients and hinder their ability to maintain adequate self-care and treatment adherence [7]. In fact, since 2010, cancer has been the leading cause of mortality on the island, surpassing even cardiovascular disease [8]. Given the disproportionate impact of cancer on the population, it is critical to understand and address the barriers contributing to this ongoing public health crisis.

Moreover, the existing literature consistently associates employment with improved psychological outcomes, citing enhanced daily structure, purpose, and social engagement as protective factors against depression [7]. Nonetheless, the relationship between employment and health is multifaceted. Although occupational engagement may lower the risk of affective disorders, it has also been linked to suboptimal dietary behaviors. Specifically, full-time employment may limit time for meal preparation and increase the reliance on processed or low-nutritional-value foods, due to work-related stress and financial constraints [9,10,11]. In socioeconomically disadvantaged settings such as Puerto Rico, these dynamics are further intensified by high poverty levels, limited access to healthy foods, and compounded caregiving responsibilities, which collectively increase the psychosocial burden for individuals with chronic illnesses, including cancer [8,12,13]. As demonstrated in various studies, there is a bidirectional relationship between nutrition and mental health [11]. For example, food insecurity has been associated with a higher prevalence of depression, anxiety, and fatigue in oncology patients [14]. Likewise, the subjective perception of economic hardship, understood as individuals’ evaluation of their income’s capacity to meet household needs, has emerged as a relevant indicator of emotional vulnerability, which is sometimes more significant than objective income [1]. This perception influences not only an individual’s emotional state but also their health-related decision-making, including diet, adherence to treatment, and self-care.

Although the role of social determinants of health has been well documented, there remains a significant gap in the literature regarding the interplay among employment status, perceived economic hardship, nutritional behaviors, and depressive symptoms in cancer populations. To bridge the gaps mentioned above, this study examined the correlations among occupational status, perceived income hardship, the consumption of foods with low nutritional value, and depressive symptomatology in a sample of cancer patients in Puerto Rico. The findings from this analysis aim to identify risk profiles and protective factors that can inform the design of integrated psychosocial and nutritional interventions.

## 2. Materials and Methods

A cross-sectional study was conducted with a sample of 334 adult cancer survivors residing in Puerto Rico. The data collected from these participants was obtained from two project data bases: “Post-Hurricane Cancer Care: Patients Needs after Hurricane Maria” and “Biopsychosocial predictors of tumor-associated inflammation and progression”, led by Castro-Figueroa, E.M, and Armaiz, G.N. at the Ponce Research Institute (PRI) in Puerto Rico. Participants were recruited from oncology clinics and support groups affiliated with the PRI and other associated entities. To be eligible to participate, individuals had to be at least 18 years old, have a confirmed cancer diagnosis and be classified as cancer survivors (i.e., not currently undergoing active treatment), and be capable of providing informed consent. Participants with severe cognitive impairments or acute psychiatric illnesses that prevented them from participating independently in the assessment process were excluded from the sample. For more information about recruitment and other methods, please refer to Castro-Figueroa et al. (2021) [15] and Peña-Vargas et al. (2022 and 2025) [12,16]. Data collection was carried out using a structured self-administered questionnaire. This instrument was designed to collect sociodemographic, economic, dietary, and emotional information from participants. The structured questionnaire used in this study was specifically developed for the aims of the research and the context of the population, drawing on the relevant literature and existing instruments used in oncology and psychosocial studies. While the questionnaire has not undergone formal psychometric validation, it was reviewed by experts in psychology, oncology, and public health to ensure its suitability, clarity, and relevance to the target population.

Sociodemographic variables included age (continuous variable), gender (male or female), BMI (continuous variable, obtained by dividing the participants’ weight in kg by their height in m^2^), academic level (ranging from not having completed primary education to having obtained a master’s or doctoral degree), employment status (full-time or part-time employee, retired, unemployed, student, disabled, or other), and annual household income (ordinal variable). For statistical analyses, employment status was re-categorized into three groups: not working, part-time, and full-time employment. This recategorized variable was used in the Spearman correlation, the Kruskal–Wallis test, the Mann–Whitney test, and regression analyses. Finally, a question was included on the subjective perception of economic sufficiency, in which participants were asked to indicate whether they considered their household income to be sufficient to cover their basic needs (yes or no).

The clinical variable included for this study was a self-reported diagnosis of depression (yes or no). Eating behavior was assessed using questions related to the frequency of the consumption of foods with low (red meats, bread and cereals, pastas, fried foods, sweets, soft drinks) and high (fruits, white meats, grains, vegetables, whole-grain products, water) nutritional values. The scoring process consisted of a scale that ranged from 1 to 5 (1 = I do not consume it; 2 = sometimes a month; 3 = sometimes a week; 4 = once a day; 5 = 2–4 times a day). Based on the responses obtained, a total score was generated that reflected an unhealthy eating pattern and another score reflecting a healthy eating pattern. A measure of perceived economic hardship was also included, formulated as a dichotomous question about whether household income was sufficient to meet daily needs.

The data in this study was analyzed using IBM SPSS Statistics, version 29. Descriptive statistics, including frequencies, percentages, means, and standard deviations, were used to characterize the sample. Cases with missing data for any variable of interest were excluded from the corresponding analyses using a complete case analysis approach. The chi-square test was used to evaluate the association between the perception of economic hardship and the presence of depression. Since this is a cross-sectional study, a causal relationship cannot be established; however, the dependence between dichotomous variables can be established using odds ratios (ORs), with a 95% confidence interval (95% CI). Spearman’s correlation coefficient test was used to explore the relationship between employment status (not working, part-time, and full-time) and the frequency of healthy and unhealthy food consumption. This test measures how one variable increases (ρ > 0) or decreases (ρ < 0) in relation to another variable. The closer the rho value (ρ) is to 1 or −1, the more significant the correlation between variables. The differences in eating habits according to the type of employment were examined using the nonparametric Kruskal–Wallis and Mann–Whitney U tests. The difference between the mean unhealthy eating scores according to the type of employment (not working, part-time and full-time) was calculated using the Kruskal–Wallis test. This test determines the difference between the mean score of three or more groups. The Mann–Whitney U test, which determines which two groups are significantly different from one another, would be used given that the Kruskal–Wallis test establishes a clinically significant difference between groups. Both tests compare groups without assuming normality. The total score in healthy and unhealthy eating was the dependent variable, while employment status was the independent variable. Finally, a logistic regression analysis was performed to identify predictors associated with depression, adjusting for variables such as educational level and age. All analyses were performed with a statistical significance level of *p* < 0.05.

## 3. Results

**Figure 1:**
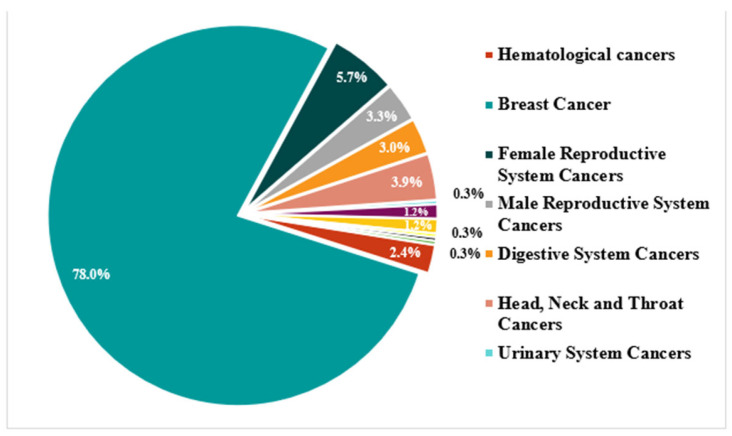
Distribution of cancer types in study sample.

The sample consisted of 334 subjects, mostly women (93%), with a mean age of 57.32 ± 12.13 years. The study sample predominantly consisted of participants diagnosed with breast cancer, which accounted for 78.0% of all cases. Other cancer types represented smaller proportions, including hematological cancers (5.7%), female reproductive system cancers (3.9%), male reproductive system cancers (3.3%), digestive system cancers (3.0%), and head, neck, and throat cancers (2.4%). Less frequent diagnoses included urinary system cancers (1.2%), bone and soft tissue cancers (1.2%), skin cancers (0.3%), respiratory system cancers (0.3%), nervous system tumors (0.3%), and other epithelial carcinomas (0.3%) (see Figure 1). Regarding the marital status of the study population, 42% of the individuals identified themselves as married, while 20% declared themselves single, and the remaining 14% identified themselves as divorced. In terms of education, 30.8% of the sample had completed secondary education, 27.2% had a bachelor’s degree, and 24% had a technical or associate degree. In addition, 24% of the participants were retired, 20% were unemployed, 20% were in full-time employment, and 18% reported being disabled, reflecting a heterogeneous distribution in terms of occupational status (see Table 1).

The statistical analyses performed revealed significant findings. First, the chi-square analysis revealed a significant association between the perception of economic hardship due to income and the presence of depression (OR = 0.54, *p* = 0.033, see Table 2). Subjects who perceived that their income was not sufficient to cover household expenses presented higher odds of having depression, which highlights the importance of subjective perceptions of economic security as a key indicator of emotional vulnerability.

A negative correlation was found between employment level and the consumption of healthy foods (ρ = −0.126, *p* = 0.022, see Table 3). On the other hand, a positive correlation was found between employment level and the consumption of unhealthy foods (ρ = 0.157, *p* = 0.015, see Table 3), suggesting that individuals with employment, especially full-time employment, tend to consume food products of high nutritional value less frequently, while also consuming food products of low nutritional value more frequently.

The Kruskal–Wallis analysis identified significant differences in the consumption of unhealthy foods according to employment type (*p* = 0.017), see Table 4. Complementarily, the Mann–Whitney test confirmed that full-time employed subjects reported a significantly higher consumption of such foods compared with unemployed subjects (*p* = 0.010), see Table 5.

Regarding mental health, the logistic regression analysis indicates that employment acts as a protective factor, reducing the likelihood of having depression (aOR = 0.694, *p* < 0.05), even after controlling for educational level and age. However, an elevated body mass index (BMI; used as a continuous variable) was found to increase the risk of depression significantly (aOR = 1.063, *p* = 0.002), see Table 6, suggesting an inverse relationship between metabolic health and emotional well-being. Having a higher income was not significantly associated with having depression.

## 4. Discussion

This study highlights the crucial role of determinants in shaping the dietary behavioral patterns and emotional well-being of cancer patients in Puerto Rico. This study’s findings reveal a significant association between employment and the perceptions of economic security with mental health outcomes. Also, a particularly relevant aspect is the paradoxical relationship between full-time employment and the consumption of foods with a high and low nutritional value. While participation in the labor market has been associated with psychological benefits, such as structured routine, a sense of purpose, and social interaction, it can also create significant barriers to maintaining healthy eating habits [7]. Such barriers can be attributed to factors such as work stress, time constraints, and the increased consumption of fast food [7]. This is often due to time constraints, fatigue, or prioritizing dietary convenience. Similar patterns have been documented in studies involving chronic illness populations, where work demands and obligations can compromise nutritional quality [17]. In the Puerto Rican context, it has been observed that food insecurity affects a significant number of cancer patients, who face structural challenges to accessing nutritious food, exacerbated by factors such as poverty and a lack of institutional support [12]. Nevertheless, despite these nutritional risks, the protective role of employment in mental health remains evident. These results are consistent with previous evidence suggesting that employment can mitigate the adverse psychosocial effects of a cancer diagnosis by providing emotional, financial, and relational stability [18].

However, it is necessary to consider the structural challenges prevailing in Puerto Rico’s employment context, where unfavorable economic conditions may mitigate the positive effects of employment on mental health. In addition, the association between an elevated body mass index and an increased likelihood of depressive symptoms confirms the findings of the scientific literature on the interaction between metabolic and emotional health. Several mechanisms have been proposed to explain the link between obesity and depression, including chronic inflammation, social stigma, and reduced self-esteem [19]. At the same time, the subjective perception of financial hardship has been shown to have a significant association with emotional well-being. This highlights the importance of evaluating not only objective socioeconomic indicators but also the lived experiences of individuals regarding economic security. It has been shown that perceived financial hardship can increase stress levels and reduce a person’s sense of control and self-efficacy. These effects may be particularly noticeable in disadvantaged settings such as Puerto Rico [20]. Similarly, research involving Latino populations demonstrated that financial strain is strongly correlated with diminished emotional, physical, and functional well-being, even after accounting for clinical and demographic variables. These findings underscore the role of perceived financial security as a key determinant of quality of life [21,22,23]. Furthermore, studies conducted in Puerto Rico reveal that social isolation and limited access to mental health services during crises exacerbate the psychological distress experienced by cancer patients [13].

Interestingly, although employment status was significantly associated with depressive symptoms, reported income was not. This finding may seem counterintuitive, especially given that socioeconomic pressures are known to affect mental health. However, it is possible that employment provides psychological and social benefits, such as routine, purpose, identity, and social interaction, that extend beyond financial compensation. In contrast, income level alone may not accurately reflect the subjective experience of economic strain or stability. Furthermore, perceived financial hardship, rather than objective income, may be more closely aligned with emotional well-being, as supported by prior research [1,20]. These results suggest that the psychosocial aspects of employment may offer greater protection against depression than economic factors alone, particularly in environments characterized by structural socioeconomic challenges.

From a clinical and psychosocial perspective, these results highlight the imperative need to adopt comprehensive intervention approaches adapted to local living conditions. In this sense, intervention strategies must consider the impact of work responsibilities on nutritional quality, as well as design programs that strengthen the perception of financial security and emotional resilience. Among the methodological limitations of this study, it is worth noting its cross-sectional design, which does not permit the establishment of causal relationships. In addition, the overrepresentation of women in the sample limits the generalizability of the findings to male cancer populations. Although the small number of male participants precluded a formal sensitivity analysis, future studies should aim to recruit more gender-balanced samples to explore potential sex-based differences in these relationships. In this study, depression status was assessed through a single self-reported yes/no question rather than a validated symptom scale, which may introduce misclassification bias and limit the comparability with studies employing standardized instruments. The possibility arises that factors not assessed, such as social support or medical comorbidity, may have influenced the results obtained. It is recommended that future studies adopt longitudinal designs to examine the evolution of these relationships over time. For instance, longitudinal studies could observe how changes in employment status influence nutritional behaviors and emotional well-being in newly diagnosed cancer patients across different treatment phases. Furthermore, intervention studies could be designed to evaluate the effectiveness of employment support programs, nutritional counseling, and financial aid services in improving dietary quality and mental health outcomes in this population. Likewise, the evaluation of the impact of multicomponent interventions, such as nutritional counseling, food subsidies, and integrated mental health services, on emotional and behavioral outcomes in oncology patients is considered vital. Prior studies have demonstrated that culturally adapted interventions, including nutrition education, personalized messaging, and community-based support groups, have resulted in significant improvements in dietary behaviors and emotional well-being among Latina breast cancer survivors [24,25]. The implementation of strategies such as financial counseling and education on insurance matters has shown promise in mitigating financial toxicity and may contribute to an improved quality of life for cancer patients [26].

One of the key strengths of this study is its focus on a socioeconomically vulnerable and underserved population: cancer patients in Puerto Rico, who are often underrepresented in psychosocial and nutritional research. By incorporating objective indicators, such as employment status, and subjective measures, such as perceived financial hardship, this study provides a more comprehensive understanding of the complex socioeconomic factors influencing emotional well-being and dietary behaviors. Furthermore, exploring modifiable factors, particularly employment, economic perceptions, and nutritional habits, offers valuable insight for designing contextually relevant, interdisciplinary interventions. This integrative approach provides valuable evidence for the literature on the social determinants of health in oncology, particularly within Latinx and populations with limited resources.

## 5. Conclusions

This study suggests that employment status and the perception of economic hardship may impact the dietary patterns and mental health of cancer patients living in Puerto Rico. Although employment has been associated with a lower probability of depressive symptoms, it also leads to a higher consumption of foods of low nutritional value, which reflects a risk factor for emotional stability and dietary risks [10]. In addition, a higher body mass index and perceived financial inadequacy are significant risk factors for depression [27]. These findings underscore the need to develop integrated and contextualized interventions that simultaneously address nutrition, emotional well-being, and socioeconomic challenges. It is recommended that future research employs longitudinal designs and evaluates psychosocial and nutritional programs tailored to the cancer population.

## Figures and Tables

**Table 1:** Sociodemographic data of study sample.

	Frequency	Percent
Marital status		
Single, never married	67	20%
Married	140	42%
Consensual union (living without getting married)	31	9%
Divorced	48	14%
Separated	11	3%
Widow	37	11%
Total	334	100%
Academic Level		
I did not finish elementary school	1	0.3%
Elementary School (up to 6 grade)	8	2.4%
Middle School (up to 9 grade)	20	6.0%
High School (up to 12 grade)	103	30.8%
Technical Course/associate degree	80	24.0%
Bachelor’s degree	91	27.2%
Master’s or Doctorate	31	9.3%
Total	334	100%
Employment Status		
Full-time	68	20%
Part-time	23	7%
Retired	81	24%
Student	1	1%
Unemployed	66	20%
Disabled	62	18%
Other	27	8%
Missing	6	2%
Total	334	100%
Approximate annual income		
Less than USD 12,000 (USD 1000 per month or less)	142	43%
USD 12,001–USD 19,000 (USD 1001–USD 1583 per month)	87	26%
USD 19,001–USD 35,000 (USD 1584–USD 2917 per month)	66	20%
USD 35,001–USD 60,000 (USD 2917–USD 5000 per month)	33	10%
USD 60,001–USD 100,000 (USD 55,001–USD 8333 per month)	5	1%
USD 100,001–USD 250,000 a year	1	0%
Total	334	100%
Perceived Economic Hardship Do you think the income coming into your home is enough to cover all expenses and needs?		
No	216	65%
Yes	118	35%
Total	334	100%

Note: “Other” in employment status variable includes homemaker, self-employed, and community leader.

**Table 2:** Association between economic hardship and depression.

	Perceived Economic Hardship	*n* (333)	Odds Ratio (95% CI)	*p*-Value
No Depression	No	156	0.54 (0.306–0.951)	0.033 *
	Yes	98		
Depression	No	59		
	Yes	20		

*n*, number of participants; CI, confidence interval; * *p*-value < 0.05.

**Table 3:** Correlation between employment status and eating habits.

Category	Employment Coding	Correlation Coefficient (ρ)	*p*-Value
Healthy eating	0 = Not working	−0.126	0.022 *
	1 = Part-time		
	2 = Full-time		
Unhealthy eating	0 = Not working	0.157	0.015 *
	1 = Part-time		
	2 = Full-time		

ρ rho value; * *p*-value < 0.05.

**Table 4:** Difference between eating habit scores (healthy and unhealthy eating) according to employment status.

Category	Employment Type	Mean Score	*p*-Value
Healthy eating	Not working	172.03	0.063
	Part-time	140.74	
	Full-time	146.28	
Unhealthy eating	Not working	155.28	0.017 *
	Part-time	187.54	
	Full-time	188.83	

* *p*-value < 0.05.

**Table 5:** Difference between unhealthy eating scores according to employment status.

Comparisons	Mean Score	*p*-Value
First Comparison		
Not working	128.19	0.110
Part-time	154.28	
Second comparison		
Not working	146.09	0.010 *
Full-time	177.08	
Third Comparison		
Part-time	45.26	0.876
Full-time	46.25	

* *p*-value < 0.05.

**Table 6:** Predictive factors associated with depression.

Category	aOR (95% CI)	*p*-Value
Body Mass Index	1.063 (1.023–1.104)	0.002 *
Income	0.860 (0.656–1.129)	0.278
Employment Status	0.694 (0.537–0.897)	0.005 *

Logistic regression used interaction terms with all three variables. All ORs were adjusted for age and education level; * *p*-value < 0.05.

## Data Availability

The data used in this study were obtained from a previously collected dataset and are not publicly available due to privacy and ethical restrictions. Requests to access the dataset should be directed to the corresponding author and will be evaluated in accordance with institutional policies and IRB approval.

## Abbreviations

The following abbreviations are used in this manuscript:

CI	Confidence Interval
*n*	Number of participants
NCI	National Cancer Institute
OR	Odds Ratio
*p*	*p* value
PR	Puerto Rico
PRI	Ponce Research Institute
ρ	Correlation Coefficient
